# Pan‐cancer proteogenomic landscape of whole‐genome doubling reveals putative therapeutic targets in various cancer types

**DOI:** 10.1002/ctm2.1796

**Published:** 2024-08-15

**Authors:** Eunhyong Chang, Su‐Jung Kim, Hee Sang Hwang, Kyu Jin Song, Kwoneel Kim, Min‐Sik Kim, Se Jin Jang, Sungyong You, Kwang Pyo Kim, Joon‐Yong An

**Affiliations:** ^1^ Department of Integrated Biomedical and Life Science Korea University Seoul Republic of Korea; ^2^ L‐HOPE Program for Community‐Based Total Learning Health Systems Korea University Seoul Republic of Korea; ^3^ Department of Applied Chemistry Institute of Natural Science Kyung Hee University Yongin Republic of Korea; ^4^ Department of Pathology University of Ulsan College of Medicine, Asan Medical Center Seoul Republic of Korea; ^5^ Department of Biomedical Science and Technology Kyung Hee Medical Science Research Institute, Kyung Hee University Seoul Republic of Korea; ^6^ Department of Biology Kyung Hee University Seoul Republic of Korea; ^7^ Department of Biomedical and Pharmaceutical Sciences Kyung Hee University Seoul Republic of Korea; ^8^ Department of New Biology DGIST Daegu Republic of Korea; ^9^ New Biology Research Center DGIST Daegu Republic of Korea; ^10^ Center for Cell Fate Reprogramming and Control DGIST Daegu Republic of Korea; ^11^ SG Medical, Inc. Seoul Republic of Korea; ^12^ Department of Urology Cedars‐Sinai Medical Center Los Angeles California USA; ^13^ Department of Computational Biomedicine Cedars‐Sinai Medical Center Los Angeles California USA; ^14^ School of Biosystem and Biomedical Science College of Health Science, Korea University Seoul Republic of Korea

Dear Editor,

Whole‐genome doubling (WGD) occurs in various cancer types and plays a crucial role in tumour development and genomic instability.^1^, ^2^ However, the proteogenomic characteristics and molecular regulators governing WGD have yet to be elucidated. By integrating large‐scale multi‐omics data from the Clinical Proteomic Tumor Analysis Consortium (CPTAC),^3^ we classified three types of WGD tumours across cancer types. This study elucidates the mutational signatures, molecular pathways, transcription factor (TF) regulation and kinase phosphorylation networks enriched in tumours with WGD to explore potential drug targets.

Our study integrated genomic, transcriptomic, proteomic and phosphoproteomic data from 1060 samples representing 10 cancer types including breast cancer (BRCA), clear cell renal cell carcinoma (CCRCC), colon adenocarcinoma (COAD), glioblastoma (GBM), high‐grade serous carcinoma (HGSC), head and neck squamous cell carcinoma (HNSCC), lung squamous cell carcinoma (LSCC), lung adenocarcinoma (LUAD), pancreatic ductal adenocarcinoma (PDAC) and uterine corpus endometrial carcinoma (UCEC) (Figure 1A). WGD was found to be prevalent in 42% of tumours, with the highest in HGSC (83%) and the lowest in PDAC (9.4%) (Figure 1B,C; Table S1A). Although no clinical phenotypes were associated with WGD status across pan‐cancer, the advanced tumour stage was linked to WGD status in HNSCC (false discovery rate [FDR] < .05) (Figure S1A,B).

**FIGURE 1:**
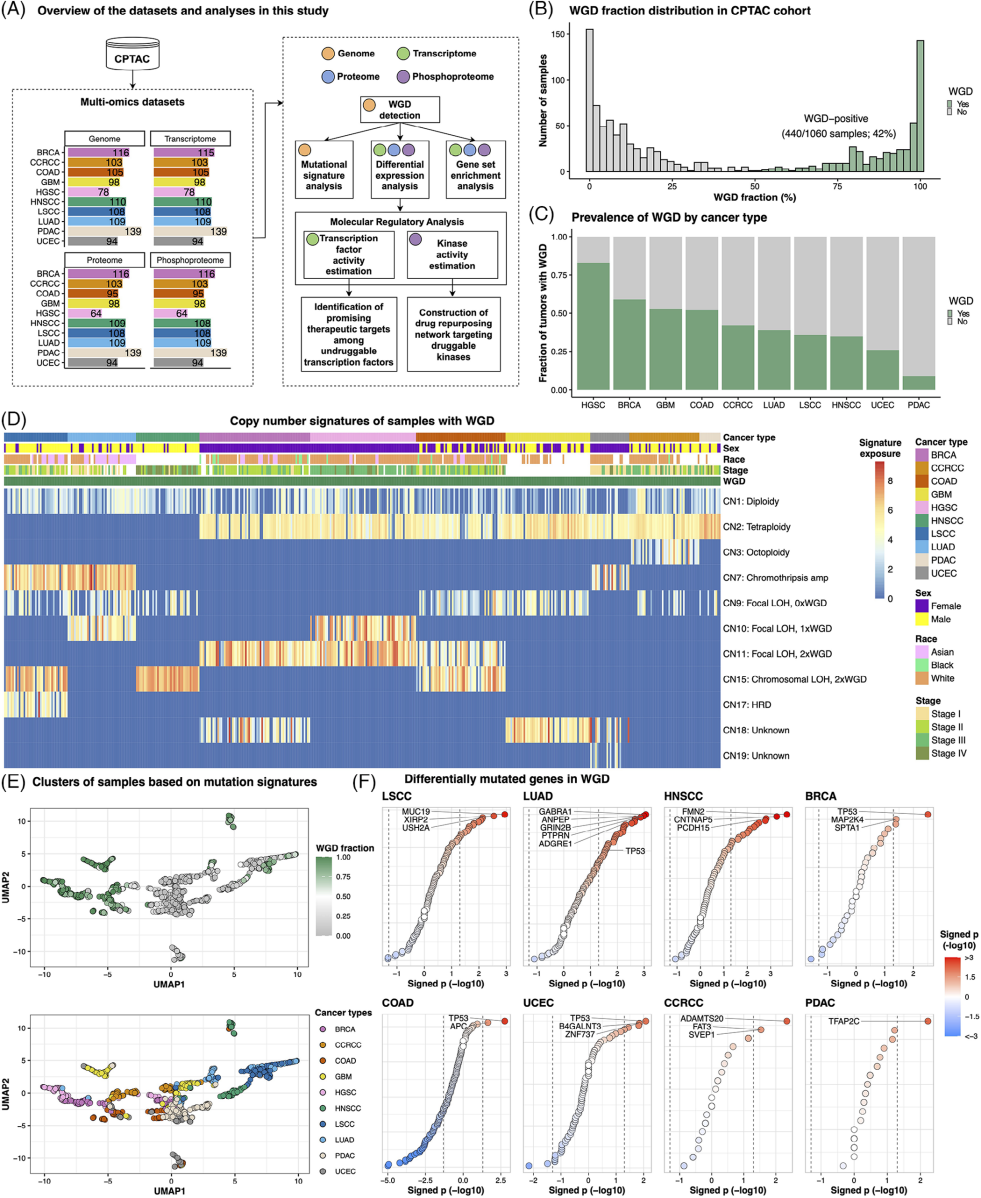
Copy number signatures associated with whole‐genome doubling (WGD). (A) Schematic representation of the multi‐omics datasets collected for each cancer type and the subsequent analysis pipeline. (B) Distribution of the WGD fraction within the Clinical Proteomic Tumour Analysis Consortium (CPTAC) cohort, displaying a bimodal pattern. (C) Prevalence of WGD by cancer type. (D) Heatmap based on copy number signature exposure values in samples with WGD. Copy number signatures were derived from the Catalogue of Somatic Mutations In Cancer (COSMIC). (E) Uniform manifold approximation and projection (UMAP) plot based on signature exposure values of single‐base‐substitution, double‐base‐substitution, indel and copy number alteration. Each dot indicates samples and is colored based on WGD fraction (top) and cancer types (bottom). (F) Dot plot depicting differentially mutated genes in WGD in each cancer type. High‐grade serous carcinoma (HGSC) and glioblastoma (GBM), which had no significantly enriched mutations in WGD, are not shown. The position of each dot along the *x*‐axis and the colour of the dots indicate signed *p*‐values obtained using Fisher's exact test. Only the top three significant genes were labelled in each cancer type, except for colon adenocarcinoma (COAD) and pancreatic ductal adenocarcinoma (PDAC), which had only two and one significant genes, respectively. In lung adenocarcinoma (LUAD), *PTPRN, GRIN2B*, *ANPEP* and *ADGRE1* genes exhibited the same *p*‐values, and *TP53* was additionally labelled. BRCA, breast cancer; CCRCC, clear cell renal cell carcinoma; HNSCC, head and neck squamous cell carcinoma; LSCC, lung squamous cell carcinoma; UCEC, uterine corpus endometrial carcinoma.

Mutation signature analyses showed that WGD was associated with specific copy number (CN) signatures (Figure 1D; Table S1B). WGD in LSCC, LUAD and HNSCC showed enrichment for either or both CN7 and CN15 signatures (FDR < .05). Since CN7 indicates chromothripsis amplification and CN15 indicates chromosomal loss‐of‐heterozygosity (LOH) with twice‐genome‐doubling,^4^ WGD in LSCC, LUAD and HNSCC may have occurred within a highly unstable genome. WGD in BRCA and HGSC was enriched for CN11 (focal LOH with twice‐genome‐doubling) (FDR < .05), suggesting that WGD in these malignancies occurred within a focally unstable genome. Many WGD samples from CCRCC, COAD, GBM, PDAC and UCEC were found to be enriched for CN2 (FDR < .05), indicating tetraploidy. Consequently, three WGD types were defined: ‘WGD type 1 (highly unstable genome)’ in LSCC, LUAD and HNSCC; ‘WGD type 2 (focal instability)’ in BRCA and HGSC; ‘WGD type 3 (tetraploidy)’ in CCRCC, COAD, GBM, PDAC and UCEC (Figure 1E).

Driver mutations associated with WGD were identified in each tumour type. Whereas previous studies suggest enrichment of *TP53* mutations in WGD tumours,^1^, ^2^ we identified enrichment of *TP53* mutations exclusively in the WGD‐positive samples of BRCA, COAD, UCEC and LUAD (*p* < .05) (Figure 1F; Table S1D). Moreover, we found that more than 80 gene amplifications resulted in gene and protein expression upregulation (involved in cell cycle and metabolism) in WGD‐positive LSCC, LUAD and HNSCC (WGD type 1) (Figure S1C; Table S1D,E). Additionally, more than 100 mutations were enriched in WGD type 1 (*p* < .05), whereas other cancer types exhibited <15 significant gene mutations associated with WGD. As a highly unstable genome has a high level of tumour mutational burden (TMB),^5^ WGD type 1 had a higher TMB in WGD‐positive than in WGD‐negative tumours (*p* < .05; Wilcoxon rank‐sum test) (Figure S1D,E). COAD exhibited lower TMB in WGD‐positive tumours than in WGD‐negative tumours (*p* < .001; Wilcoxon rank‐sum test) and *TP53* and *APC* mutations were augmented in WGD‐positive COAD (*p* < .05; Fisher's exact test) (Figure 1F). Genomic instability is therefore a catalyst for WGD in LSCC, LUAD and HNSCC, whereas *TP53* and *APC* mutations may serve as primary drivers of WGD in COAD.

Pathway analysis revealed distinct biological processes enriched in WGD tumours across different cancer types (Figure 2A; Table S2A,B). Although previous studies have reported upregulation of the cell cycle and downregulation of immune response pathways in WGD,^1^, ^2^, ^6^ this pattern was specific to WGD type 1 (Figure 2B,C). WGD type 2 tumours were enriched for the dTTP metabolism pathway, which is responsible for DNA synthesis and maintenance, and the DNA endoreduplication pathway, which is a known mechanism inducing WGD^7^ (Figure S2A,B). WGD‐positive COAD demonstrated significant activation of the Wnt signalling pathway, putatively attributed to *APC* mutation^8^ (Figure S2C).

**FIGURE 2:**
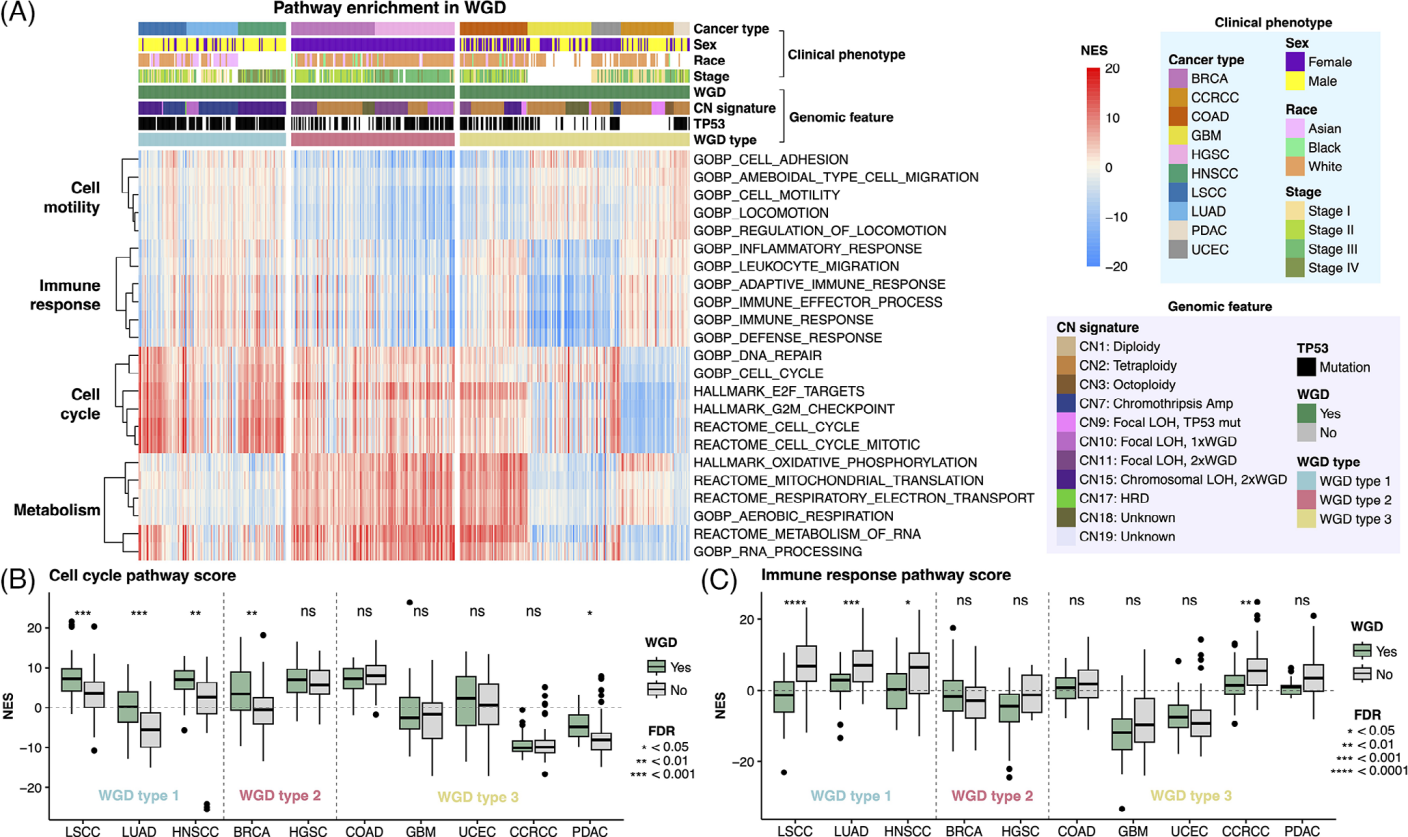
Pathway enrichment in whole‐genome doubling (WGD)‐positive tumours. (A) Normalised enrichment scores (NES) of pathways related to cell motility, immune response, cell cycle and metabolism in WGD‐positive tumours. Pathways exhibiting significance (FDR < .05) and a log fold‐change > .25 are shown. (B and C) Boxplot comparing NES scores of cell cycle (HALLMARK_E2F_TARGETS) and immune response pathways (GOBP_IMMUNE_RESPONSE) between WGD‐positive and WGD‐negative tumours in individual cancer types.

To identify a potential therapeutic target for WGD tumours, TF activity was measured using the gene expression profile of WGD tumours. E2F family and MYC TFs were activated in pan‐cancer WGD tumours, particularly in WGD type 1 (FDR < .1) (Figure 3A,B; Table S3A,B). Since many TFs have been deemed as ‘undruggable’,^9^ we developed an integrative framework incorporating protein expression, cancer dependency and patient survival data to prioritise WGD‐activated TFs as therapeutic targets (Figure 3C). Hence, BPTF was identified as a putative target for WGD‐positive HNSCC tumours with poor prognosis (Figure 3D–F). Potential targets for WGD were found: GLI2 (LSCC), TFDP1 and TFDP2 (LSCC, HNSCC, PDAC and HGSC), E2F3 (LUAD) and SREBF2 (BRCA) (Figure S3A–I; Table S3C). We further validated that the knockdown of E2F3 in WGD‐positive LUAD cell lines, and BPTF, SFPQ and REST in WGD‐positive HNSCC cell lines resulted in a greater reduction in viability relative to WGD‐negative cell lines (Figure 3G).

**FIGURE 3:**
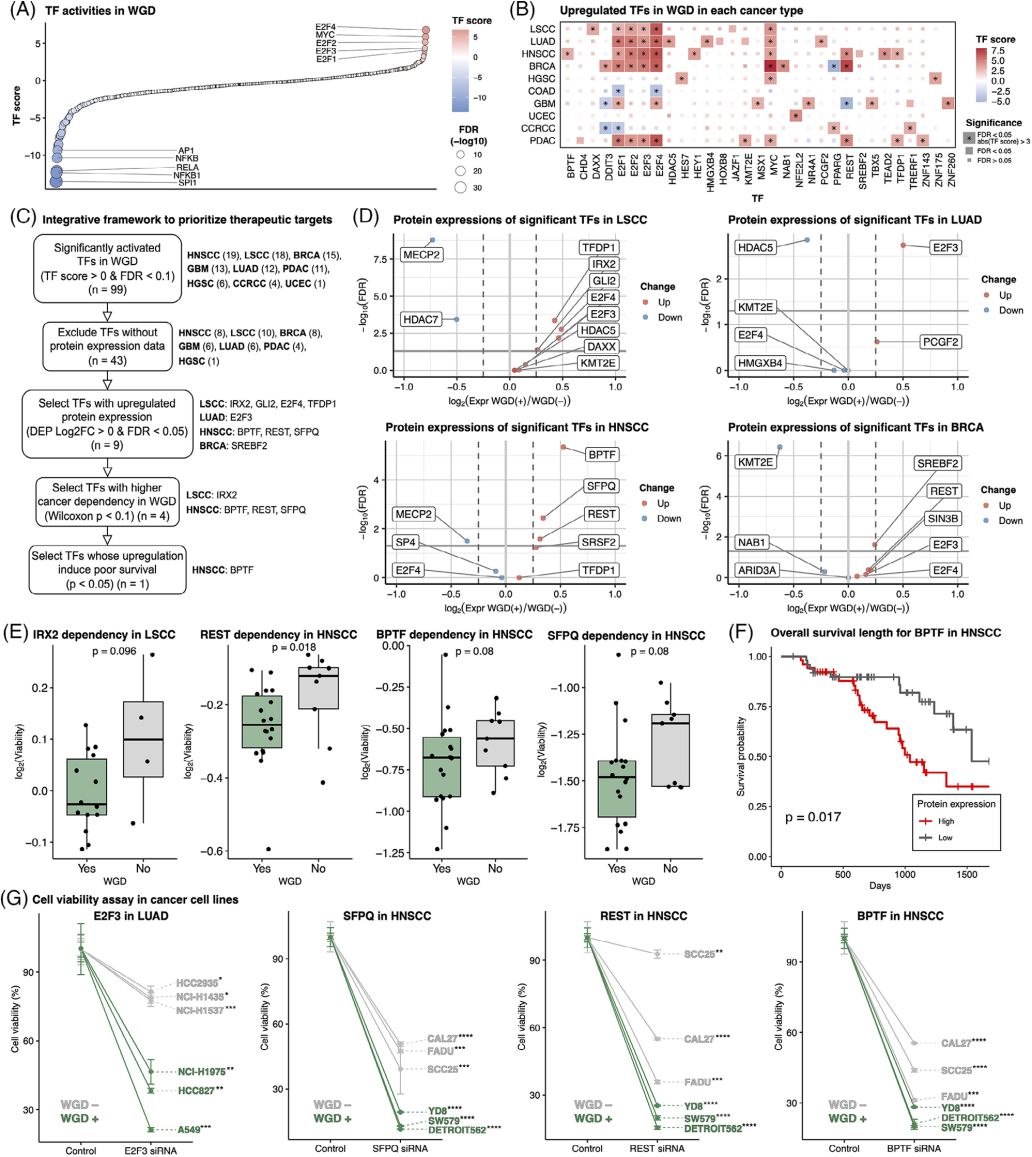
Determining potential therapeutic targets among activated transcription factors (TFs) in whole‐genome doubling (WGD). (A) Dot plot depicting estimated TF scores in WGD‐positive versus WGD‐negative tumours across pan‐cancer analysis. The top and bottom five TFs, ranked by TF score, are labelled. (B) Significantly upregulated TFs in WGD in each tumour type. The tiles are coloured based on the TF estimation score; TFs meeting the criteria of FDR < .05 and TF score > 3 are denoted with asterisks. (C) Schematic diagram showing the filtering processes to identify effective therapeutic targets. The number of TFs or the name of TFs remaining after each step is indicated on the right. (D) Volcano plots of differentially expressed proteins among significant TFs in WGD‐positive tumours versus WGD‐negative tumours. (E) Boxplots comparing log_2_ cell viability between WGD‐positive and WGD‐negative cells after depleting genes with CRISPR in each cancer cell line. (F) Kaplan–Meier curve of overall survival based on protein expression levels of BPTF in head and neck squamous cell carcinoma (HNSCC). (G) Cell viability assay for functional validation of identified TF targets. Specifically, E2F3 in lung adenocarcinoma (LUAD) and BPTF, REST and SFPQ in HNSCC were tested as these TFs were identified as promising targets and were experimentally available. Point indicates mean viability and error bar indicates standard deviation. *p*‐Values from paired *t*‐tests are denoted with asterisks. ^*^ *p* < .05, ^**^ *p* < .01, ^***^ *p* < .001 and ^****^ *p* < .0001.

As kinases are well‐known druggable proteins,^9^ we estimated kinase activity using proteomics and phosphoproteomics profiles of WGD tumours and constructed a drug‐repurposing network targeting kinases upregulated in WGD. We observed cancer‐type‐specific activations in WGD tumours, including CDK1/2 in cell cycle regulation, CSNK2A1 in DNA damage response, PAK4 in proliferation and apoptosis suppression, and PRKAA2/ULK1 in hypoxia‐induced autophagy (Figure 4A,B; Table S4A). This indicates that WGD accelerates cell proliferation during tumourigenesis but also induces DNA damage and hypoxia, necessitating kinase activation to inhibit apoptosis. The drug repurposing network analysis suggested potential drugs with significant selectivity in WGD, such as palbociclib (LSCC, BRCA), erlotinib, gefitinib and neratinib (LSCC) and nintedanib (PDAC) (Figures 4C and S4G–I; Table S4C).

**FIGURE 4:**
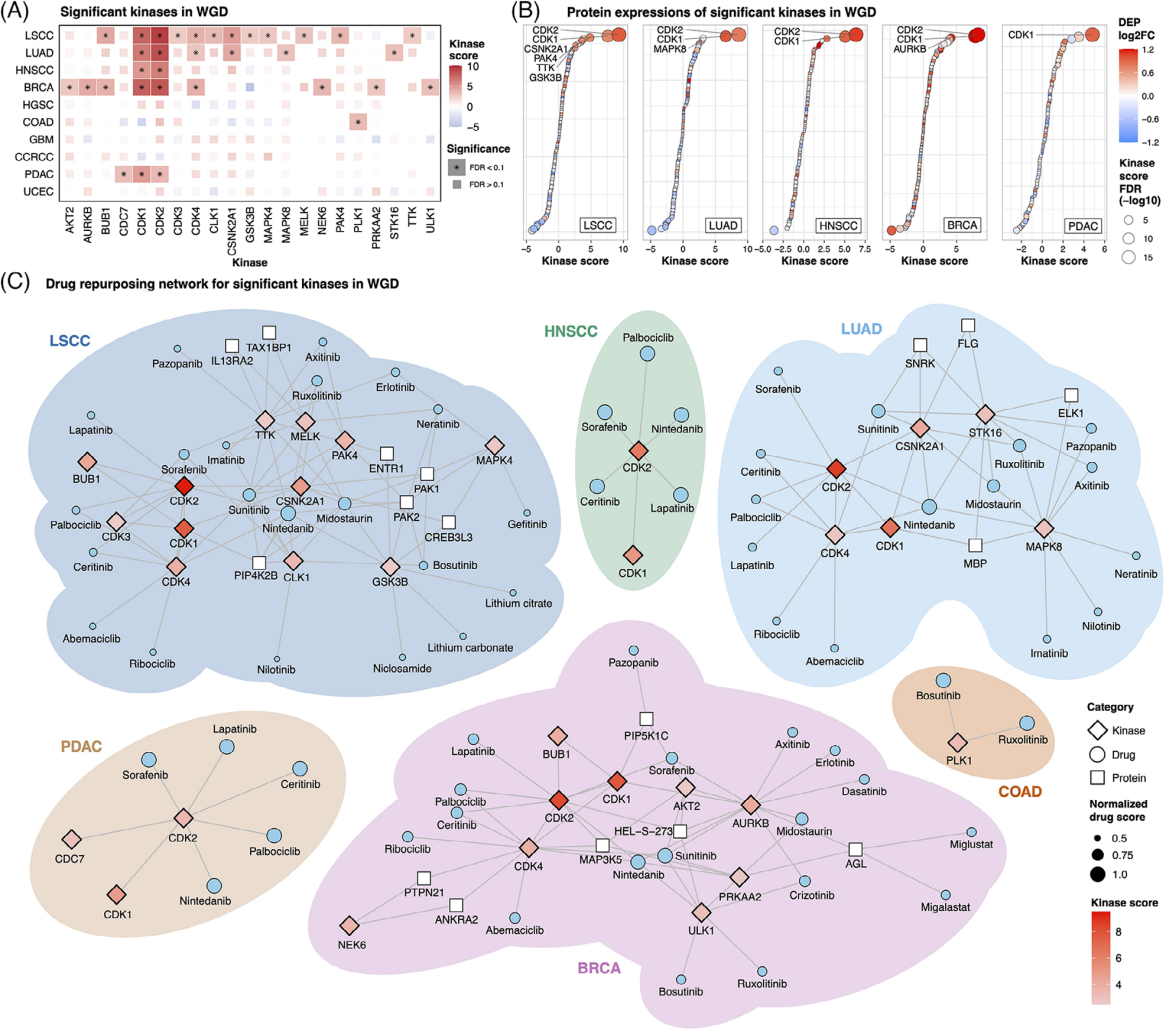
Drug repurposing strategies to target key kinases in whole‐genome doubling (WGD). (A) Kinases with significant upregulation of expression in association with WGD in each tumour type. The tiles are coloured based on the kinase estimation score and kinases showing statistically significant upregulation are denoted with asterisks. (B) Dot plot depicting differentially expressed proteins among significantly upregulated kinases in WGD‐positive tumours versus WGD‐negative tumours. Six cancer types with significant kinase activation in WGD are displayed (lung squamous cell carcinoma [LSCC], lung adenocarcinoma [LUAD], head and neck squamous cell carcinoma [HNSCC], breast cancer [BRCA], pancreatic ductal adenocarcinoma [PDAC] and colon adenocarcinoma [COAD]), excluding COAD, which had no kinases with significantly upregulated protein expression. Kinases with significant upregulation of protein expression (FDR < .05 and log_2_ fold‐change > 0), as well as activation (FDR < .1), have been labelled. (C) Drug repurposing network for key kinases associated with WGD in each tumour type. Six cancer types with significant kinase activation of WGD are shown (LSCC, LUAD, HNSCC, BRCA, PDAC and COAD).

In conclusion, our study presents the first proteogenomic analysis to characterise the molecular features of WGD across various cancer types. Three WGD types were defined: WGD type 1 (highly unstable genome), WGD type 2 (focal instability) and WGD type 3 (tetraploidy). Cell cycle pathway activation and immune response pathway inactivation were specific to WGD type 1. The integrative framework characterised a comprehensive molecular network from gene regulation to kinase–phosphorylation activity and suggested BPTF as a putative target for WGD‐positive HNSCC. Further studies are warranted to evaluate the efficacy of drugs targeting WGD‐specific TFs and kinases.

## AUTHOR CONTRIBUTIONS

Eunhyong Chang and Joon‐Yong An designed the study. Eunhyong Chang analysed the data. Su‐Jung Kim conducted cell viability assay. Eunhyong Chang, Su‐Jung Kim, Hee Sang Hwang, Kyu Jin Song, Kwoneel Kim, Min‐Sik Kim, Se Jin Jang, Kwang Pyo Kim, Sungyong You and Joon‐Yong An wrote and reviewed the manuscript. All the authors approved the final version of the manuscript.

**[** **dataset]** Li Y, Dou Y, Da Veiga Leprevost F, et al. CPTAC dataset. 2023. https://www.linkedomics.org/data_download/; https://doi.org/10.1016/j.ccell.2023.06.009

## CONFLICT OF INTEREST STATEMENT

Kwang Pyo Kim is the CEO of NioBiopharmaceuticals, Inc. Se Jin Jang is the Chief Technology Officer of SG Medical, Inc. All other authors report no conflicts of interest.

## ETHICS STATEMENT

Not applicable.

## Supporting information

Supporting Information

Supporting Information

Supporting Information

Supporting Information

Supporting Information

## Data Availability

All CPTAC datasets we used are publicly available. Genome and transcriptome data were downloaded from the GDC data portal (https://portal.gdc.cancer.gov). Global proteomic and phosphoproteomic data were downloaded from LinkedOmics (https://www.linkedomics.org/). All data generated during this study are included in Supporting Information.
