# Supplementary material for: Pan‐cancer proteogenomic landscape of whole‐genome doubling reveals putative therapeutic targets in various cancer types
Source: Clin Transl Med. 2024 Aug 15;14(8):e1796. doi: 10.1002/ctm2.1796 (PMC11327001; doi:10.1002/ctm2.1796)
Supplement: Supplementary file 1 — Supporting Information [file CTM2-14-e1796-s005.docx]

**Methods**

**Data collection and preprocessing**

We obtained genomic, transcriptomic, global proteomic, and phosphoproteomic data from the Clinical Proteomic Tumor Analysis Consortium. The mutation annotation format (MAF) file for each sample and the segment-level copy number variant (CNV) text file for the samples in CPTAC phase-3 (comprising CCRCC, GBM, HNSCC, LSCC, LUAD, PDAC, and UCEC) were downloaded from the GDC data portal (<https://portal.gdc.cancer.gov>). For gene-level CNV data, we downloaded “CNV_WashU_v1” from PDC (<https://pdc.cancer.gov/pdc/cptac-pancancer>). RNA-seq data were downloaded from the GDC data portal. Global proteomic and phosphoproteomic data were downloaded from LinkedOmics (<https://www.linkedomics.org/>).

To standardize the global protein and phosphoprotein data across various cancer types, we followed the methodology from previous papers from CPTAC.^1-3^ Briefly, we normalized the global protein and phosphoprotein data using z-scores. Then, global proteins and phosphoproteins with > 30% missing values in each cancer-type sample were excluded. The remaining missing values were imputed via k-nearest neighbor imputation with k=5 implemented through the R package impute v1.74.1 (<https://doi.org/10.18129/B9.bioc.impute>).

**CNV calling and WGD detection**

As segment-level CNV data for BRCA, COAD, and HGSC were unavailable, we acquired BAM files from the GDC data portal and performed the CNV calling process. FACETS v0.16.0 ^4^ was employed to discern allele-specific copy number (CN) information. The input for FACETS consisted of paired tumor-normal BAM files and a VCF file containing common and germline polymorphic sites downloaded from <https://www.ncbi.nlm.nih.gov/variation/docs/human_variation_vcf/>.

Using major CN and minor CN data derived from segment-level CNV data, we defined samples as “WGD-positive” if over 50% of their autosomal genome exhibited a major CN greater than or equal to two as described in Bielski et al.^5^

**Identification of clinical phenotypes associated with WGD status**

We performed a multiple linear regression analysis to determine which clinical phenotypes are associated with WGD status. The clinical metadata available in CPTAC datasets, including age, sex, race, stage, smoking, and alcohol consumption, were all tested. The multiple linear regression used the following formula:

$$WGD status \sim Age+Sex+Race+Stage+Smoking+Alcohol$$

The analysis was conducted for both pan-cancer and individual cancer types. For HGSC, BRCA, and UCEC, which consist exclusively of female samples, sex was excluded from the formula. Furthermore, due to the unavailability of certain data, stage for GBM, race for HNSCC, smoking for BRCA, COAD, HGSC, and UCEC, and alcohol for BRCA, COAD, and HGSC were excluded from the formula for the respective cancer type analyses.

**Mutational signature analysis and WGD type classification**

To decipher cancer type-specific patterns of WGD, we conducted a mutational signature analysis on single-base-substitution (SBS), double-base-substitution (DBS), indel (ID), and CNV using the COSMIC signature database v3 ^6^ and R package Sigminer v2.1.5 ^7^. Non-negative matrix factorization was employed to determine the number of signature groups or factorization ranks. This involved creating a tumor-by-component matrix with 50 runs and checking the ranks ranging from 2 to 12. Each signature was identified using the COSMIC signature with the highest cosine similarity. Subsequently, hierarchical clustering was performed, and the samples were assigned to one of the signatures based on the consensus matrix. Then, we integrated exposure values from SBS, DBS, ID, and CNV, and performed UMAP clustering these values using R package umap v0.2.10.0 ^8^. The parameters were set with a seed of 42, n_neighbors of 10, n_components of 2, and the metric as “euclidean”.

For WGD type classification, we assigned each cancer type’s WGD a representative CN signature according to the majority of CN signatures observed in WGD-positive samples. LSCC, LUAD, and HNSCC were grouped into WGD type 1, as they showed enrichment in either or both CN7 (Chromothripsis amplification) and CN15 (Chromosomal LOH and twice-genome-doubling). Despite their differences, both signatures share the characteristic of WGD occurring against a background of chromosomal instability, and this similarity was supported by their spatial proximity in the UMAP clustering. Additionally, we assigned WGD in BRCA and HGSC to WGD type 2. Although the majority of WGD-positive BRCA samples exhibited CN2 (tetraploidy), the exposure value of CN11 (focal LOH and twice-genome-doubling) was greater than CN2 and showed spatial proximity to HGSC on the UMAP plot. Other cancer types were assigned to WGD type 3 representing CN2, as most WGD-positive samples in these cancers exhibited CN2.

**Driver mutation and TMB analysis**

To identify which small mutations are enriched in WGD-positive tumors, we utilized R package maftools v2.20.1 ^9^. We integrated each sample’s MAF files downloaded from the GDC data portal and compared samples with and without WGD using the “mafCompare” function. This function performs Fisher’s exact test between the two groups for each gene.

To determine which gene-level CNVs are associated with WGD status, we conducted linear regression with the following model:

$$CN \sim WGD.status$$

The p-value was obtained from the linear regression, and the adjusted p-value was calculated using the Benjamini–Hochberg adjustment.

As more than 100 mutations were enriched in WGD type 1, we hypothesized that this might be due to a high level of tumor mutation burden (TMB). Therefore, we calculated TMB by variants per megabase (Mb) using whole exome sequencing data.

**Analysis of differential expression**

To discern the differentially expressed genes between WGD-positive and WGD-negative samples, we employed the “DESeq” function from the DESeq2 R package v1.40.2 ^10^. The raw count data were used for the generation of a DESeqDataSet using the “DESeqDataSetFromMatrix” function. Following a variance stabilizing transformation and the exclusion of data points with a mean raw count less than 50, we conducted the differential expression analysis utilizing the “DESeq” function, which is grounded in the negative binomial distribution. The DEG analysis was performed using the following formula:

$$design = \sim WGD.status$$

To detect the differential expression of global and phospho-proteins in the WGD samples, we employed an integrated hypothesis testing method following the methodology proposed by Hwang et al ^11^. Briefly, we performed t-tests, median difference tests, and Wilcoxon tests and combined the p-values from the statistical tests using Stouffer's method. This approach enhances the accuracy of identifying true-positive data elements compared with traditional methods when applied to biological data.

**Gene set enrichment analysis**

We conducted a gene set enrichment analysis to elucidate the activated biological pathways in each sample. The “zscore” function from the R package GSVA v1.48.3 ^12^ was utilized for analyzing transcriptome, global proteome, and phospho-proteome data. Pathways from various databases, including hallmark (“h.all.v2023.1.Hs.symbols.gmt.txt”), KEGG (“c2.cp.kegg.v2023.1.Hs.symbols.gmt.txt”), Reactome (“c2.cp.reactome.v2023.1.Hs.symbols.gmt.txt”), GO (“c5.go.v2023.1.Hs.symbols.gmt.txt”), and Wikipathway (“c2.cp.wikipathways.v2023.1.Hs.symbols.gmt.txt”) sourced from MSigDB were incorporated into the gene set variation analysis. Subsequently, we performed a t-test between WGD-positive tumors and WGD-negative tumors to identify significantly regulated pathways in the context of WGD.

**Estimation of transcription factor and kinase activity**

To identify therapeutic targets for WGD-positive tumors, we sought to determine the activated transcription factors (TF) and kinases in WGD. For estimating TF activity, we used differentially expressed genes (DEGs) and the CollecTRI ^13^ database, which provides a TF-target interaction prior knowledge network. For kinase activity estimation, we utilized differentially expressed phosphoproteins and the OmniPathR ^14^ database, which contains a kinase-substrate prior knowledge network. These data inputs were analyzed using a multivariate linear model (MLM) as follows:

$$Y= \beta X+\psi$$

where the dependent variable Y represents the log-2-fold change from differential expression analysis, and the independent variable X is the connectivity matrix representing the associations with TFs or kinases. Ψ denotes the normally distributed error of the fit, and β represents the scores of the TF or kinase activity. In the decoupleR R package v2.6.0 ^15^, we used the “run_mlm” function with the minimum size of regulons set to 1. The p-value was obtained from the multivariate linear model, and the adjusted p-value was calculated using the Benjamini–Hochberg adjustment.

**Integrative framework for therapeutic target identification**

We developed an integrative framework incorporating protein expression, cancer dependency, and patient survival data to prioritize WGD-activated TFs and kinases as therapeutic targets. Among significantly activated TFs and kinases in WGD (false discovery rate [FDR] < 0.1), we first excluded those without protein expression data and selected TFs and kinases with significantly upregulated protein expressions.

As cancer dependency is a crucial aspect of being an effective therapeutic target, we compared the cancer dependencies between WGD-positive and WGD-negative cells, using data downloaded from DepMap (<https://depmap.org/portal/>). Specifically, we used “CRISPR (DepMap Public 23Q2+Score, Chronos),” “RNAi (Achilles+DRIVE+Marcotte, DEMETER2),” and “Aneuploidy” data for each cancer cell line. We filtered the cell lines based on annotated genome doubling statuses, classifying cell lines with one or more instances of genome doubling as WGD-positive, and those without genome doubling as WGD-negative. A one-sided Wilcoxon rank-sum test was conducted to determine whether WGD-positive cells exhibited lower viability compared to WGD-negative cells.

To further refine our target identification, we also assessed whether the targets were associated with patient survival. Using the R package survival v3.5.7 ^16^, we performed survival analysis via the Kaplan–Meier estimation model. The survival duration of the patients and death events were used as inputs for the analysis. To identify the TFs and kinases associated with poor prognosis, we compared survival curves between samples with the top 50% and bottom 50% protein expression for each significantly activated TF and kinase in WGD (FDR < 0.1).

These findings are shown in **Figure S3** and **Figure S4A-F**. Additionally, we annotated the TF or kinase score, copy number, and druggability on these TFs and kinases. Druggability was confirmed from DGIdb (v5.0.5) ^17^. TFs or kinases lacking interactions with drugs were deemed “undruggable,” those with interactions with FDA-approved drugs were designated as “FDA-approved,” and those interacting with known drugs but not with FDA-approved ones were labeled as “druggable.”

**Target validation experiment**

We conducted a cell viability assay for functional validation of identified TF targets. Specifically, we targeted E2F3 in LUAD and BPTF, REST, and SFPQ in HNSCC as these TFs were identified as promising targets in our integrative framework and were experimentally available.

Based on the WGD status of each cancer cell line,^18^ we selected HCC827, NCI-H1975, and A549 for WGD-positive LUAD and HCC2935, NCI-H1435, and NCI-H1573 for WGD-negative LUAD cell lines. For WGD-positive HNSCC, DETROIT562, SW579, and YD8 were selected and for WGD-negative HNSCC, CAL27, SCC25, and FADU were selected. HCC2935, HCC827, NCI-H1975, A549, CAL27, SCC25, SW579, and DETROIT562 were obtained from the ATCC (Manassas, Virginia, USA). NCI-H1435, NCI-H1573, FADU, and YD8 were obtained from the Korea Cell Line Bank (Seoul, Korea).

All LUAD cell lines, FADU, and YD8 were maintained in RPMI 1640 cell culture media. The culture medium for the CAL27 was Dulbecco’s modified Eagle medium (DMEM) with 10% FBS. SCC25 cells were cultured in a 1:1 mixed medium of DMEM and Ham's F-12 Nutrient Mixture, supplemented with 10% FBS. DETROIT562 cells were grown according to the manufacturer's specification using DMEM supplemented with 10% FBS. SW579 cells were cultured in an L15 medium supplemented with 10% FBS. All culture media were supplemented with 10% (v/v) heat-inactivated fetal bovine serum, 100 U/mL penicillin, and 100 g/mL streptomycin. All cell lines were grown in a sterile, humidified incubator at 37 °C with 5% CO_2_ and passaged every 3–5 days, depending on the cell line, to maintain appropriate cell densities.

The cell viability was determined using the 3-(4,5-dimethylthiazol-2-yl)-2,5-diphenyltetrazolium bromide (MTT) reduction assay. MTT was purchased from Sigma Chemical Co. (St. Louis, MO, USA). Human-specific E2F3-siRNA (sense 5’-UACUAGUCUUCUGUAUCUGTT-3’; antisense 5’-CAGAUACAGAAGACUAGUATT-3’), BPTF-siRNA (sense 5′-GAGGAAAGGCCUUCGAUCATT-3′; antisense 5′-UGAUCGAAGGCCUUUCCUCTT-3′), REST-siRNA (sense 5′-GUGACUACCAGAACUCGAATT-3′; antisense 5′-UUCGAGUUCUGGUAGUCACTT-3′), SFPQ-siRNA (sense 5′-GAACAAAUGAGGCGCCAAATT-3′; antisense 5′-UUUGGCGCCUCAUUUGUUCTT3′), and negative control siRNA were purchased from Bioneer, Inc. (Daejeon, South Korea). Three technical replicates were tested for LUAD cell lines, and four were tested for HNSCC cell lines. Cells were transfected with siRNA using transfection reagents (Lipofectamine RNAiMAX; Invitrogen, Carlsbad, CA, USA). After incubation, cells were treated with the MTT solution (final concentration, 1 mg/ml) for 2 h. The dark blue formazan crystals formed in intact cells were dissolved with dimethyl sulfoxide and the absorbance at 570 nm was read using a microplate reader. Results were expressed as the percentage of MTT reduction obtained in the treated cells, assuming that the absorbance of control cells was 100%.

**Drug repurposing network analysis**

As kinases are well-known druggable targets, we sought to identify effective drugs for WGD-positive tumors. To achieve this, we constructed the drug repurposing network using DrugSt.One (v1.2.0).^19^ Significantly upregulated kinases (FDR < 0.1) in the WGD for each tumor type were used as inputs for the search algorithms. NeDRex (v2.21.0) was used for protein-protein and protein-drug interaction searches. For the protein-protein interaction network, we used a multi-Steiner algorithm, setting the number of trees to five, tolerance to five, and hub penalty to 0.5. For the protein-drug interaction network, we used a harmonic centrality algorithm, setting the hub penalty to 0.5 and the result size to 50 and excluding indirect and non-approved drugs.

**Drug sensitivity analysis**

To evaluate the drugs featured in the drug repurposing network, we compared drug sensitivities between WGD-positive and WGD-negative cells. “PRISM Repurposing Public 24Q2”, “Drug sensitivity AUC (Sanger GDSC1)”, and “Drug sensitivity AUC (CTD^2)” data for each cancer cell line were downloaded from DepMap (<https://depmap.org/portal/>). We conducted a one-sided Wilcoxon rank-sum test to assess whether the WGD-positive cells exhibited higher drug sensitivity than the WGD-negative cells.

**Limitations**

Despite our comprehensive analysis, there are limitations within our study. For cancer types included in CPTAC phase 2 (BRCA, COAD, and HGSC), whole-exome sequencing data were utilized to infer WGD, a method potentially less precise than whole-genome sequencing. Additionally, the varying proportion of samples in each cancer stage may affect the frequency of WGD-positive samples and WGD type classification for each cancer type. Larger scale proteogenomic investigations are warranted to fully understand WGD characteristics in various cancers.

**References**

1. Gillette MA, Satpathy S, Cao S, et al. Proteogenomic Characterization Reveals Therapeutic Vulnerabilities in Lung Adenocarcinoma. *Cell*. Jul 9 2020;182(1):200-225 e35. doi:10.1016/j.cell.2020.06.013

2. Satpathy S, Krug K, Jean Beltran PM, et al. A proteogenomic portrait of lung squamous cell carcinoma. *Cell*. Aug 5 2021;184(16):4348-4371 e40. doi:10.1016/j.cell.2021.07.016

3. Krug K, Jaehnig EJ, Satpathy S, et al. Proteogenomic Landscape of Breast Cancer Tumorigenesis and Targeted Therapy. *Cell*. Nov 25 2020;183(5):1436-1456 e31. doi:10.1016/j.cell.2020.10.036

4. Shen R, Seshan VE. FACETS: allele-specific copy number and clonal heterogeneity analysis tool for high-throughput DNA sequencing. *Nucleic Acids Res*. Sep 19 2016;44(16):e131. doi:10.1093/nar/gkw520

5. Bielski CM, Zehir A, Penson AV, et al. Genome doubling shapes the evolution and prognosis of advanced cancers. *Nat Genet*. Aug 2018;50(8):1189-1195. doi:10.1038/s41588-018-0165-1

6. Tate JG, Bamford S, Jubb HC, et al. COSMIC: the Catalogue Of Somatic Mutations In Cancer. *Nucleic Acids Res*. Jan 8 2019;47(D1):D941-D947. doi:10.1093/nar/gky1015

7. Wang S, Li H, Song M, et al. Copy number signature analysis tool and its application in prostate cancer reveals distinct mutational processes and clinical outcomes. *PLoS Genet*. May 2021;17(5):e1009557. doi:10.1371/journal.pgen.1009557

8. McInnes L, Healy J, Melville J. Umap: Uniform manifold approximation and projection for dimension reduction. *arXiv preprint arXiv:180203426*. 2018;

9. Mayakonda A, Lin D-C, Assenov Y, Plass C, Koeffler HP. Maftools: efficient and comprehensive analysis of somatic variants in cancer. *Genome research*. 2018;28(11):1747-1756.

10. Love MI, Huber W, Anders S. Moderated estimation of fold change and dispersion for RNA-seq data with DESeq2. *Genome Biol*. 2014;15(12):550. doi:10.1186/s13059-014-0550-8

11. Hwang D, Rust AG, Ramsey S, et al. A data integration methodology for systems biology. *Proceedings of the National Academy of Sciences*. 2005;102(48):17296-17301.

12. Hänzelmann S, Castelo R, Guinney J. GSVA: gene set variation analysis for microarray and RNA-seq data. *BMC bioinformatics*. 2013;14:1-15.

13. Muller-Dott S, Tsirvouli E, Vazquez M, et al. Expanding the coverage of regulons from high-confidence prior knowledge for accurate estimation of transcription factor activities. *Nucleic Acids Res*. Nov 10 2023;51(20):10934-10949. doi:10.1093/nar/gkad841

14. Turei D, Korcsmaros T, Saez-Rodriguez J. OmniPath: guidelines and gateway for literature-curated signaling pathway resources. *Nat Methods*. Nov 29 2016;13(12):966-967. doi:10.1038/nmeth.4077

15. Badia-i-Mompel P, Vélez Santiago J, Braunger J, et al. decoupleR: ensemble of computational methods to infer biological activities from omics data. *Bioinformatics Advances*. 2022;2(1):vbac016.

16. Therneau T. A package for survival analysis in S. *R package version*. 2015;2(7):2014.

17. Cannon M, Stevenson J, Stahl K, et al. DGIdb 5.0: rebuilding the drug-gene interaction database for precision medicine and drug discovery platforms. *Nucleic Acids Res*. Jan 5 2024;52(D1):D1227-D1235. doi:10.1093/nar/gkad1040

18. Cohen-Sharir Y, McFarland JM, Abdusamad M, et al. Aneuploidy renders cancer cells vulnerable to mitotic checkpoint inhibition. *Nature*. Feb 2021;590(7846):486-491. doi:10.1038/s41586-020-03114-6

19. Maier A, Hartung M, Abovsky M, et al. Drugst. One-A plug-and-play solution for online systems medicine and network-based drug repurposing. *Arxiv*. 2023;

**Supplementary figures**

**
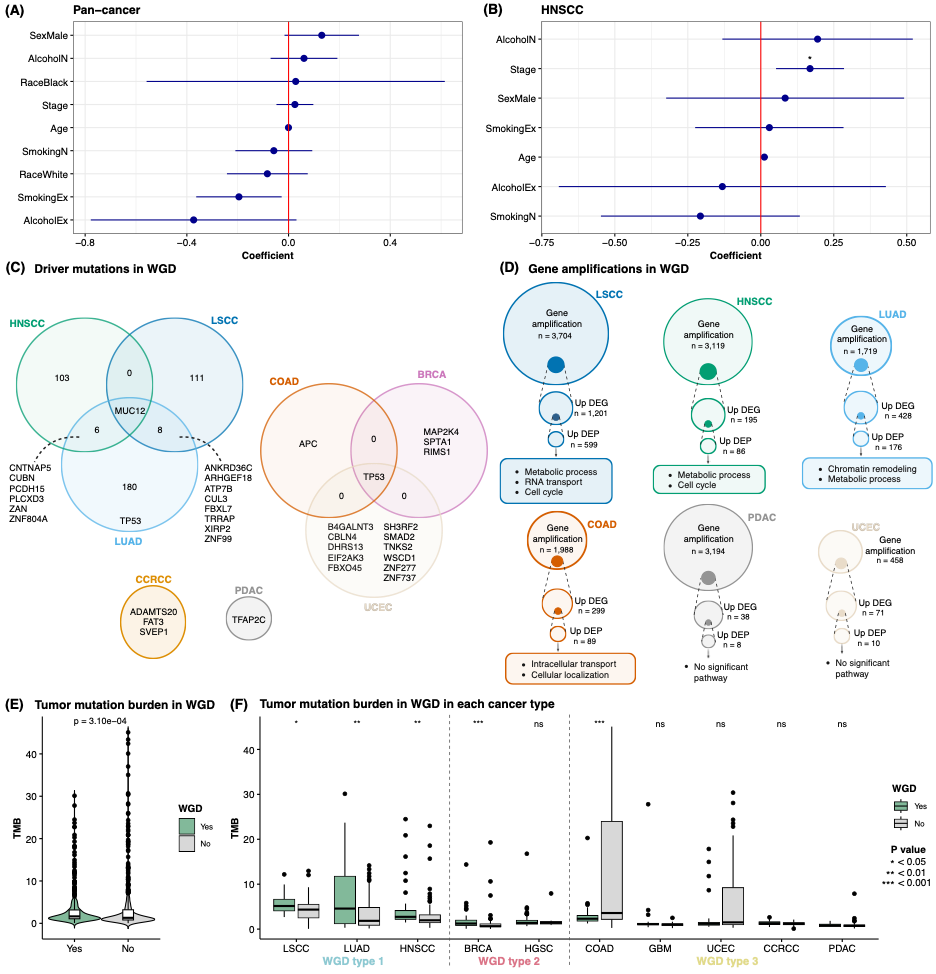
**

**Figure S1. Clinical phenotype association and tumor mutation burden in WGD**

**(A-B)** Associations between WGD status and clinical phenotypes in pan-cancer (A) and in HNSCC (B). Sex, Race, Smoking, and Alcohol data were tested as categorical variables, and Stage and Age were tested as continuous variables. *FDR < 0.05 (Multiple linear regression). **(C)** The number of small mutations significantly enriched in WGD in each cancer type (p < 0.05). There were no significantly enriched genes in HGSC and GBM. **(D)** The number of significant gene amplifications (FDR < 0.1), upregulated DEGs (FDR < 0.05), and upregulated DEPs (FDR < 0.05) in WGD in each cancer type. Representative terms from non-parametric GSEA performed on genes meeting all significance criteria are shown in the box. Plots for BRCA, HGSC, GBM, and CCRCC are not shown as there were no significant gene amplifications in these cancer types. **(E)** Violin plot comparing tumor mutation burden between WGD-positive and WGD-negative tumors across 10 cancer types. **(F)** Boxplots comparing tumor mutation burden between WGD-positive and WGD-negative tumors within each cancer type.


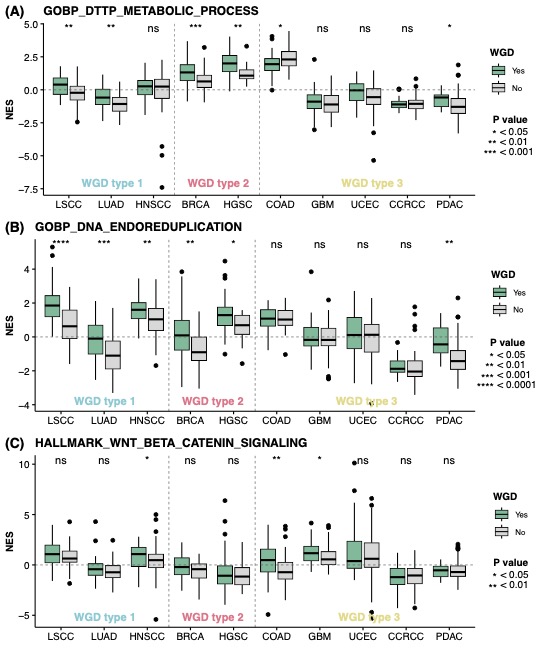


**Figure S2. Pathway enrichment in WGD-positive tumors**

**(A-C)** Boxplot comparing Normalized enrichment scores (NES) score of dTTP metabolism pathway (GOBP_DTTP_METABOLIC_PROCESS), DNA endoreduplication pathway (GOBP_DNA_ENDOREDUPLICATION), and Wnt signaling pathway (HALLMARK_WNT_BETA_CATENIN_SIGNALING) between WGD-positive and WGD-negative tumors in individual cancer types.

**Figure S3. Activated TFs in WGD in each cancer type**

**(A-I)** Significantly upregulated TFs (FDR < 0.1) in WGD in each cancer type are shown. Significant features are indicated as an asterisk (TF score, FDR < 0.05; Copy number amplification, FDR < 0.1; DEG, FDR < 0.05; DEP, FDR < 0.05; CRISPR, p < 0.1; RNAi, p < 0.1; Prognosis, p <0.05).

**Figure S4. Activated kinases and drug sensitivities in WGD**

**(A-F)** Significantly upregulated kinases (FDR < 0.1) in WGD in each cancer type are shown. Significant features are indicated as an asterisk (Kinase score, FDR < 0.05; Copy number amplification, FDR < 0.1; DEG, FDR < 0.05; DEP, FDR < 0.05; CRISPR, p < 0.1; RNAi, p < 0.1; Prognosis, p <0.05). **(G-I)** Comparison of drug sensitivities between WGD-positive and WGD-negative tumor cells using PRISM Repurposing Public 24Q2, Sanger GDSC1, and CTD^2 datasets, respectively. The size and color indicate the p-value from the Wilcoxon rank-sum test.
